# Thin silicon via crack-assisted layer exfoliation for photoelectrochemical water splitting

**DOI:** 10.1016/j.isci.2021.102921

**Published:** 2021-07-30

**Authors:** Yonghwan Lee, Bikesh Gupta, Hark Hoe Tan, Chennupati Jagadish, Jihun Oh, Siva Karuturi

**Affiliations:** 1 Department of Electronic Materials Engineering, Research School of Physics, The Australian National University, Canberra, ACT 2601, Australia; 2 Convergence Materials Research Center, Gumi Electronics and Information Technology Research Institute (GERI), Gumi 39171, Republic of Korea; 3 Australian Research Council Center of Excellence for Transformative Meta-Optical Systems, Research School of Physics, The Australian National University, Canberra, ACT 2601, Australia; 4 Department of Materials Science and Engineering, Korea Advanced Institute of Science and Technology (KAIST), Daejeon 34141, Republic of Korea; 5 Research School of Engineering, The Australian National University, Canberra, ACT 2601, Australia

**Keywords:** Chemistry, Electrochemistry, Electrochemical energy conversion, materials science, materials application, energy materials

## Abstract

Silicon (Si) has been widely investigated as a feasible material for photoelectrochemical (PEC) water splitting. Compared to thick wafer-based Si, thin Si (<50 μm thickness) could concurrently minimize the material usage allowing the development of cost-effective and flexible photoelectrodes for integrable PEC cells. This work presents the design and fabrication of thin Si using crack-assisted layer exfoliation method through detailed optical simulations and a systematic investigation of the exfoliation method. Thin free-standing Si photoanodes with sub-50 μm thickness are demonstrated by incorporating a nickel oxide (NiO_x_) thin film as oxygen evolution catalyst, light-trapping surface structure, and a rear-pn^+^ junction, to generate a photo-current density of 23.43 mA/cm^2^ with an onset potential of 1.2 V (vs. RHE). Our work offers a general approach for the development of efficient and cost-effective photoelectrodes with Si films with important implications for flexible and wearable Si-based photovoltaics and (opto)electronic devices.

## Highlights

• Design and fabrication of thin Si photoanode using crack-assisted layer exfoliation
• A systematic investigation of the crack-assisted layer exfoliation method
• Optical simulation on the dependence of photoelectrochemical performance on Si thickness
• Demonstration of thin Si photoanode with notable photoelectrochemical performance

## Graphical abstract

**Figure:**
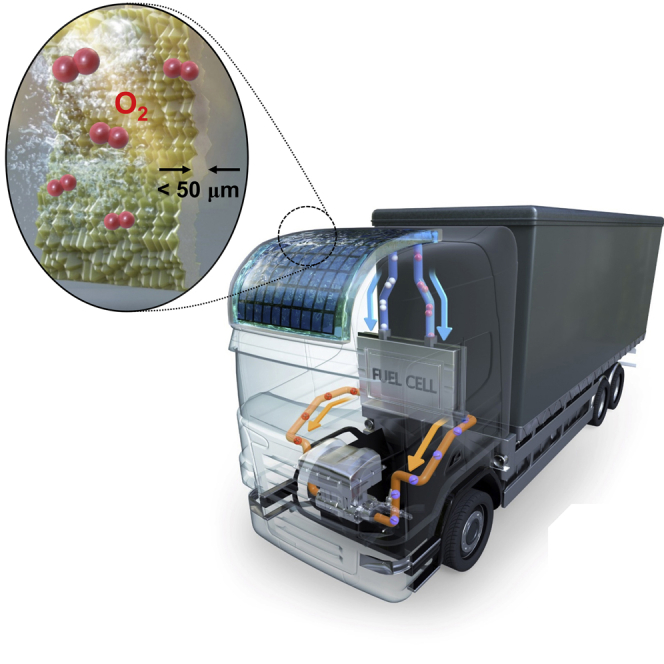

## Introduction

Photoelectrochemical (PEC) water splitting provides a promising way to convert intermittent solar irradiation into storable chemical fuels such as hydrogen (H_2_) (Lewis and Nocera, 2006; Sharma and Ghoshal, 2015). Designing advanced semiconductor photoelectrodes and obtaining a deeper understanding of their PEC performance are crucial for constructing highly efficient, stable and low-cost PEC cells for practical water splitting. Crystalline silicon (c-Si) has been considered as one of the promising materials for PEC water splitting due to its narrow bandgap (*E*_g_ = 1.1 eV) that allows absorption of visible region of the solar spectrum, appropriate band edge positions for water splitting reaction, and availability of mature manufacturing technologies (Chu et al., 2017; Luo et al., 2019). With these advantages, numerous reports appeared on wafer-based c-Si-based photoelectrodes with a thickness of 200–600 μm (Bae et al., 2015; Cai et al., 2017; Chen et al., 2015, 2017; Karuturi et al., 2020; Luo et al., 2019). Neverthless, the relatively high cost of c-Si substrate and modest performance achieved for Si photoelectrodes remained as impediments for cost-effective PEC water splitting for practical application (Jeong et al., 2013; Jung et al., 2015; Powell et al., 2012). In addition, the use of thicker wafers imparts rigidness to photoelectrodes making them unusable where flexibility is required.

**Figure 1:**
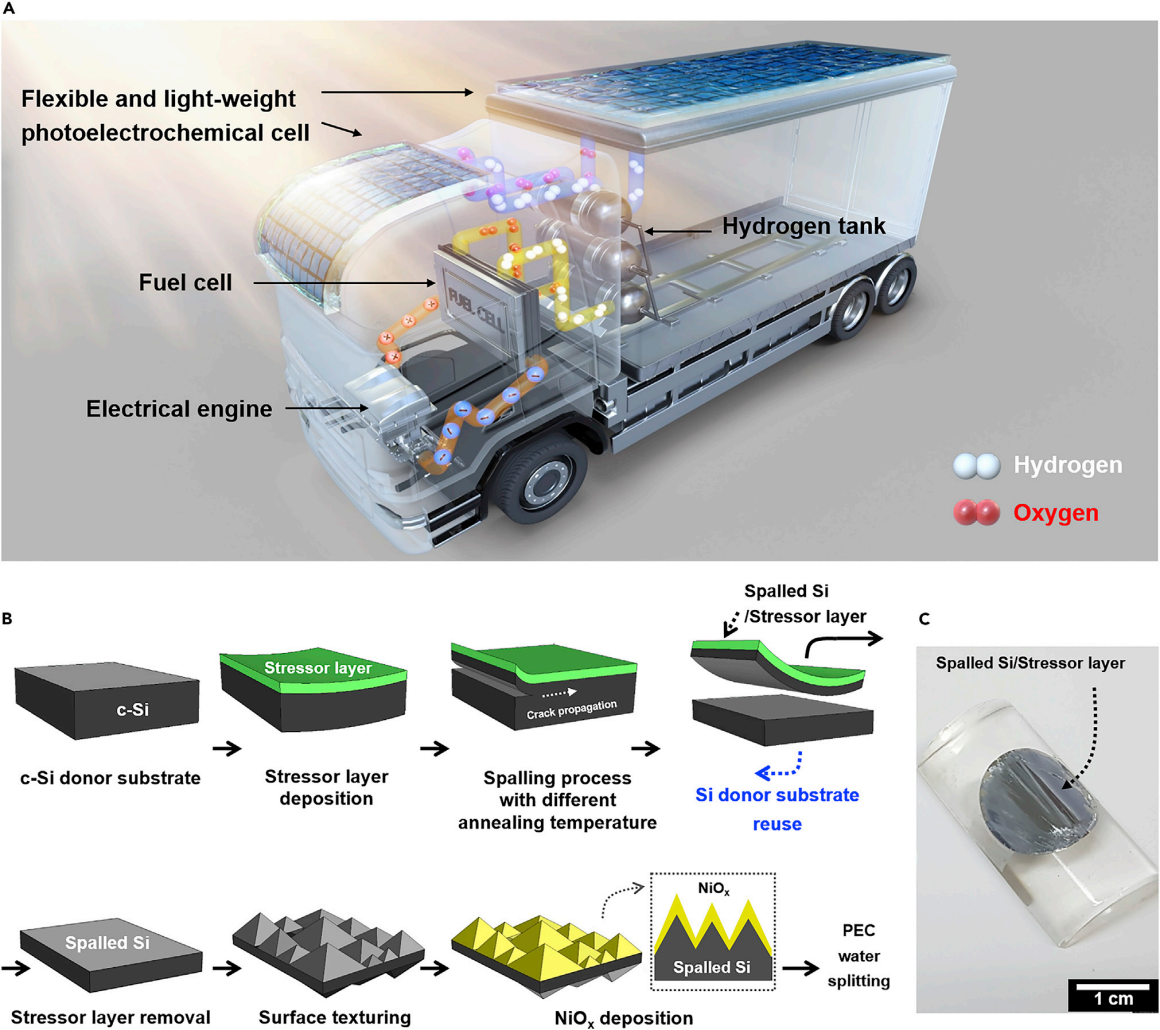
Illustration of integrated PEC cells on curved surfaces and the fabrication procedure for flexible Si photoelectrodes (A) Schematic illustration of integrated PEC water splitting cells based on flexible photoelectrodes for self-powered hydrogen vehicles. (B) Schematic illustration of the fabrication procedure for flexible Si photoanodes using the spalling process. (C) Optical image of ~8 μm-thick flexible spalled Si fabricated by controlled spalling process with 15 μm-thick Ni stressor layer.

One of the promising ways for realizing low-cost PEC-based water splitting with high performance is through utilization of thin (<50 μm) Si for the design of photoelectrodes, thereby reducing the volume of c-Si material by an order of magnitude and promoting enhanced carrier collection (Jung et al., 2015; Lee et al., 2018b; Liu et al., 2020; Massiot et al., 2020; Zhang et al., 2017). In addition to cost reduction, the use of thin Si films offers additional advantages: (1) The performance of the solar energy conversion devices becomes less affected by the material quality (*e*.*g*., bulk minority carrier lifetime) (Jeong et al., 2013), (2) enhanced photovoltage can be realized due to photo-generated carrier concentration effect from reduced Si thickness (Brendel and Queisser, 1993; Zhang et al., 2014), (3) the total weight of the device can be lowered, and (4) the high flexibility and impact resistance enables integration on irregular surfaces such as electric vehicles, ships, aeroplanes, and drones (see Figure 1A). In spite of these noticeable advantages, it remained a challenge to fabricate thin Si-based photoelectrodes while maintaining high energy conversion performance, and thus the research on Si films for PEC water splitting is seldom reported.

Various approaches such as chemical etching, epitaxial growth using chemical vapor deposition, and epi-free lift-off and transfer have been developed to fabricate Si thin films with thicknesses ranging from a few to tens of micrometers (Gordon et al., 2009; He et al., 2019; Xue et al., 2020). While the chemical etching process is not cost effective as the films are produced by etching away thick wafers, the other processes such as epitaxial gowth and lift-off are cumbersome and are energy, equipment, and cost intensive. Recently, intended cracking on brittle semiconductor substrates, known as spalling, has been introduced as a cost- and time-efficient approach for realizing wafer-scale flexible devices (Bedell et al., 2012, 2013; Kim et al., 2013; Lee et al., 2020a, 2020b; Shahrjerdi et al., 2013; Zhai et al., 2012). The spalling process enables exfoliation of semiconductor films (*i*.*e*., spalled films) from a thick target donor substrate without any material loss by controlling the crack propagation direction in the donor substrate using a tensile-stressed film, called a stressor layer. Thin c-Si has been successfully obtained *via* the spalling process and has been used in fabricating flexible electronic devices and solar cells (Ahn et al., 2017; Li et al., 2017; Weisse et al., 2012). The spalling process proceeds through crack prograpation assisted by either an applied external force or thermal annealing. Although various experimental studies have been performed to understand the spalling regimes, very little is known about the material quality obtained by these processes, particulary for PEC applications.

Here, we demonstrate free-standing thin Si photoanodes obtained using the spalling process. Optical simulations are carried out to reveal the effect of Si thickness and surface texturing on the PEC performance of spalled Si photoanodes. We systematically investigate the spalling process toward achieving a desirable thickness and material properties of spalled Si for application as a photoanode. Thin Si photoanodes are constructed by coating nickel oxide (NiO_x_) catalyst on spalled Si to achieve an excellent photocurrent density of 23.4 mA/cm^2^ with an *E*_onset_ of 1.2 eV, with the inclusion of surface texturing and rear pn^+^ junction. Finally, we discuss future opportunities to improve the PEC performance of these thin Si photoanodes.

## Results

### Preparation of thin Si photoanodes via spalling

Figure 1B illustrates the processing steps involved in the fabrication of a Si film using the spalling method and its adoption into a flexible photoanode for PEC water splitting application (see the experimental section for more details). First, Ni is electrodeposited on a c-Si donor substrate to form a tensile stress-inducing stressor layer. A Si film is then exfoliated from the c-Si donor substrate. Figure 1C shows a thin Si obtained via the spalling method. It is noted that the left-over Si donor substrate can be reused to produce additional Si films with repeated spalling steps. Thus, this approach could enable significant cost reduction for Si-based devices, in addition to imparting superior device properties such as flexibility and improved electrical performance. Subsequently, free-standing spalled Si can be obtained by removing the Ni stressor layer using a wet etching process. Surface texturing of free-standing spalled Si is performed using KOH solution to form micrometer-sized random pyramid structure. To adopt spalled Si layers into flexible photoanodes, NiO_x_, known as an efficient and transparent oxygen evolution reaction (OER) catalyst, is deposited on the front side of the textured spalled Si using sputter deposition technique. Ga-In alloy is coated on the rear side of the textured spalled Si to form a rear Ohmic contact, which is then connected to a copper wire for electrical contacting. The rear side surface is then shielded using a chemical-resistant epoxy. The final device structure thus consists of a fully functional photoanode for driving the OER under sunlight exposure.

### Design of thin Si photoanodes

**Figure 2:**
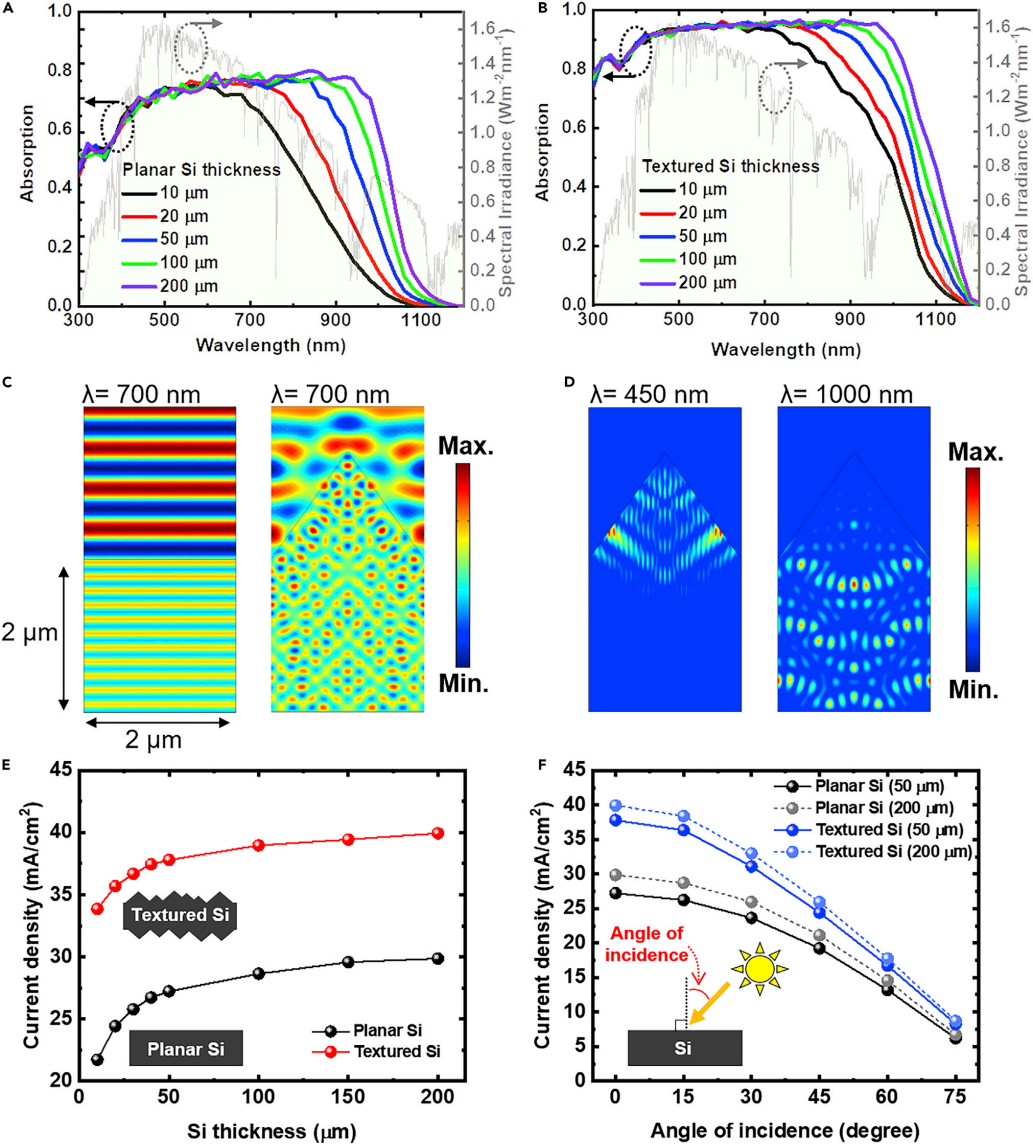
Optical properties of spalled Si films as a function of thickness with and without surface texturing (A and B) Calculated optical absorption of Si film with (A) planar surface and (B) textured surface as a function of thickness. AM1.5G spectral irradiance is also shown for reference. (C) Electric field distribution on planar surface (left) and textured surface (right) at the wavelength of 700 nm. (D) Absorption profile of textured Si at the wavelength of 450 nm (left) and 1000 nm (right). The calculated photo-generated current density of Si films as a function of (E) thickness and and (F) angle of incidence, for both planar and textured films.

While the use of Si films for photoelectrodes introduce advantages such as flexibility, cost-reduction, and reduced charge transfer lengths, it could also compromise the light harvesting ability as Si is an indirect bandgap semiconductor needing thicker material (>500 μm thickness) to fully absorb sunlight. To reveal the effect of thickness and role of texturing on light harvesting ability of Si photoanodes, we first carried out optical simulations on planar and textured Si as a function of thickness with water as a surrounding medium (see experimental section for more details). The geometry used for simulation is shown in Figure S1. Figures 2A and 2B show the calculated optical absorption (solid line) as a function of Si thickness with and without surface texturing, respectively. The optical absorption of textured Si is much higher than that of planar Si due to reduced optical reflection by light scattering effect (see Figures 2C and S2A). The optical absorption at shorter wavelengths (<700 nm) is not affected by Si thickness. However, there is significant reduction in optical absorption at longer wavelengths (>700 nm) with decreasing Si thickness. Figure 2D illustrates the dependence of light absorption on incident light wavelength in textured Si. At 450 nm, light absorption mainly occurs at the front surface (see Figure 2D, left), whereas the absorption extends deeper at 1000 nm, due to low absorption coefficients of Si at longer wavelengths (see Figures 2D and S2B). Surface texturing helps to both improve the optical absorption at longer wavelengths and reduce the optical reflection of Si. For instance, light absorptions for 50 μm-thick planar Si at 900 nm wavelengths are 64.4%, while much higher corresponding values of 91.1% are observed for textured Si of the same thickness.

To understand the effect of Si thickness on photoanode performance, we calculated the photo-generated current density with and without texturing as shown in Figure 2E. The photo-generated current density reduces gradually with decreasing thickness for both planar and textured Si due to insufficient absorption depths at longer wavelengths. Textured Si shows significantly enhanced photo-generated current density than planar Si for all thicknesses. For instance, the simulated current densities for 50 μm-thick spalled Si with and without texturing are 37.9 and 27.3 mA/cm^2^, respectively, representing a significant enhancement in current generation (∼40%) after texturing. Figure 2F shows the influence of the incident angle of light on photo-current density. Textured Si shows superior photo-generated current density than planar Si at all angles (0-75°). Therefore, these results confirm that thin Si photoanodes (20-50 μm) could achieve sufficient light harvesting when double-sided surface texturing is introduced. In addition, transparent metal oxide cocatalyst films such as NiO_x_ with appropriate thickness could further enhance the photo-generated current density due to their anti-reflection property (Sun et al., 2015).

### Fabrication of thin Si via the spalling process

We utilized the spalling process to exfoliate the Si film for photoanodes. In the spalling process, a semiconductor film can be exfoliated from a donor substrate via subsurface crack propagation assisted by either an annealing process or an applied external force. However, the role of the annealing process on layer exfoliation and its influence on the materials properties of spalled semiconductors remain unclear. With the aim of producing high quality thin Si for application as free-standing photoelectrodes, we systematically investigated the spalling process focusing on stressor layer thickness and annealing temperature.

**Figure 3:**
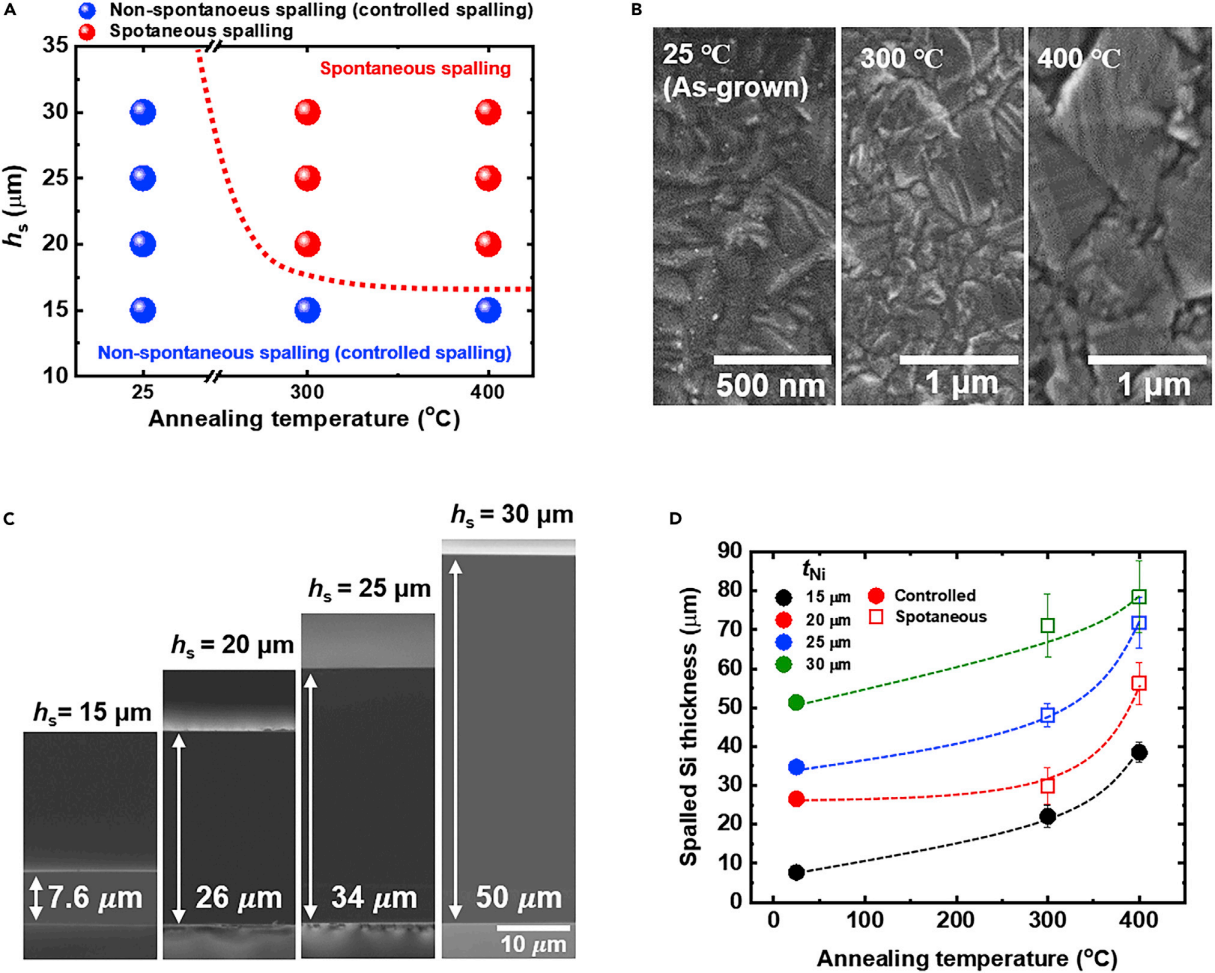
Investigation of the effect of stressor layer thickness and annealing temperature on the spalling process (A) Type of spalling process as a function of the annealing temperature and stressor layer thickness. (B) Top view SEM images of a 10 μm-thick Ni stressor layer after annealing at different temperatures. (C) Cross-sectional SEM images of spalled Si after stressor layer removal. The spalled Si films were fabricated by controlled spalling process at room temperature with different Ni stressor layer thicknesses. (D) Measured spalled Si thickness as a function of stressor layer thickness and annealing temperature. Data points represent mean ± standard deviation.

Figure 3A shows the type of spalling as a function of the annealing temperature and stressor layer thickness. It is observed that the spalling process occurs spontaneously when an appropriate thickness of stressor layer and sufficient annealing temperature are used, and we define the spalling condition as spontaneous spalling (red dots in Figure 3A). The annealing process impacts the residual stress *σ*_s_ in the Ni stressor layer. Figure 3B shows the top view scanning electron microscope (SEM) images of the Ni stressor layer before and after the annealing process. The nano-sized grains of the electrodeposited Ni layer are enlarged after annealing, which increases the tensile stress in the Ni stressor layer (Abadias et al., 2018; Wang et al., 2004). Figure S3 shows the measured *σ*_s_ in the Ni stressor layer as a function of the annealing temperature using X-ray diffraction sin^2^(*Ψ*) technique (Noyan and Cohen, 2013). The tensile *σ*_s_ in as-deposited Ni stressor layer of 403 MPa increases to 526 and 687 MPa when annealed at 300°C and 400°C, respectively.

Suo et al. developed a theoretical model on the spalling process of a bilayered system (Suo and Hutchinson, 1989). For crack propagation in the donor substrate, the energy release rate *G* has to be higher than the critical energy release rate *G*_c_ (*e*.*g*., *G*_c_ for Si is 6.4 J/m^2^) (Callister and Rethwisch, 2013). The following simplified equation shows the dependence of *G* on *σ*_s_ and stressor layer thickness (*h*_s_) in a bilayered system (Lee et al., 2016; Suo and Hutchinson, 1989).

(Equation 1) $$G\propto \sigma_{s}^{2}h_{s}$$

Therefore, if *G* increases to a value higher than *G*_c_ of c-Si donor substrate due to an increased *σ*_s_ after annealing, it results in spontaneous spalling as shown in Figure 3A.

In the case of non-spontaneous spalling (denoted by blue dots in Figure 3A), the spalling process does not occur due to insufficient *G*. Bedell et al. demonstrated that the *G* in a bilayered system can be increased to *G*_c_ by applying external mechanical force, which enables the spalling process non-spontaneously to occur even at room temperature (Bedell et al., 2013). This process is defined as controlled spalling.

We investigated the thickness of the spalled Si as a function of *h*_s_. Figure 3C shows the cross-sectional SEM images of the spalled Si fabricated by controlled spalling at room temperature. The resulting spalled thicknesses are 7.6, 26, 34, and 50 μm corresponding to *h*_s_ values of 15, 20, 25, and 30 μm, respectively. In addition, we found that the thickness of the spalled Si gradually increases with increasing annealing temperature (see Figure 3D). For instance, the thicknesses of spalled Si with 15 μm-thick Ni stressor layer are 7.6, 22, and 38.5 μm with annealing temperatures of 25°C, 300°C, and 400°C, respectively. The thickness of spalled Si, which is fixed by crack propagation depth in the spalling process, is determined by the dimensional and elastic parameters of the stressor layer (Suo and Hutchinson, 1989). For instance, the crack propagation depth is proportional to stressor layer thickness as shown in Figure 3C (Bedell et al., 2013; Lee et al., 2016; Suo and Hutchinson, 1989). In addition, we anticipate that the annealing process induces a variation of Young’s modulus of the electrodeposited Ni stressor layer. Torrents et al. showed that Young’s modulus of electrodeposited Ni thin film increases from 165 to 240 GPa after annealing at 227°C (Torrents et al., 2010). As shown in Figure 3B, the nano-crystalline grain growth in the Ni stressor layer after the annealing process increases the Young’s modulus of the Ni stressor layer, which further increases the crack propagation depth (Lee, 2018; Suo and Hutchinson, 1989). Consequently, the annealing temperature and stressor layer thickness have to be carefully optimized to obtain the desired thickness of spalled Si.

### Material properties of spalled Si

**Figure 4:**
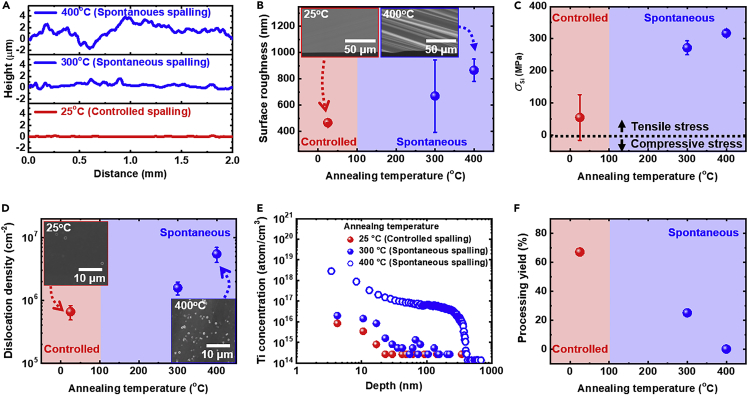
Characterization of material properties of free-standing 50 μm-thick spalled Si (A–D) (A) Surface profile, (B) surface roughness, (C) residual stress, and (D) dislocation density on the fracture surfaces of spalled Si fabricated at different annealing temperatures. The insets in (B) show the tilted-view SEM images of the fracture surface of spalled Si exfoliated via controlled spalling at room temperature (red box) and spontaneous spalling process at 400°C (blue box). The insets in (D) show the top-view SEM images of the fracture surface of spalled Si after Secco etching process. Data points represent mean ± standard deviation. (E) Measured concentration of Ti impurity in the spalled Si after annealing and removal of the Ni stressor layer. (F) Processing yield of spalled Si after stressor layer removal.

We investigated the material properties of the free-standing ∼50 μm-thick spalled Si fabricated via either the controlled or spontaneous spalling process. The controlled spalling process was conducted at room temperature (25°C) with 30 μm-thick Ni stressor layer, while the spontaneous spalling process was conducted at annealing temperatures of 300°C and 400°C with 25 and 20 μm-thick stressor layers, respectively, to produce ∼50 μm-thick spalled Si layers. Figure 4A shows the measured surface profile of the fracture surfaces of the spalled films. The spalled Si fabricated by controlled spalling at room temperature shows a relatively smooth fracture surface with an average surface roughness (*R*_a_) of 466 ± 10 nm (see Figure 4B). On the other hand, the spalled Si fabricated by spontaneous spalling process shows a much rougher fracture surface (see inset in Figure 4B), at 669 ± 275 and 865 ± 85 nm, for the 300°C and 400°C samples, respectively.

Figure 4C shows the measured residual stress *σ*_Si_ of the spalled Si films as a function of the annealing temperature. *σ*_Si_ obtained for the 25°C, 300°C, and 400°C samples are 54, 271, and 317 MPa, respectively. We removed the Ni stressor layer from the spalled Si prior to *σ*_Si_ measurements. Therefore, the measured *σ*_Si_ of the free-standing spalled Si is induced by surface defects generated during the crack propagation step (Lee et al., 2018). For more detailed analysis, we measured the defect density on fracture surfaces of spalled Si after Secco etching process (see Figure 4D) (d'Aragona, 1972; Fathi, 2007). The observed defect densities after Secco etching are 0.66×10^6^, 1.58×10^6^, and 5.46×10^6^ cm^−2^ for the 25°C, 300°C, and 400°C samples, respectively. The defect density of spalled Si follows the same trend with the measured *σ*_Si_ as a function of annealing temperature. Our observations confirm that surface roughness, *σ*_Si_, and defect density at the fracture surface of spalled Si increase dramatically when spalling is conducted at higher temperatures.

We believe that the dependence of material quality on the annealing temperature originates from a change in crack propagation velocity. In the controlled spalling process conducted at room temperature, the spalling process is carried at a timescale of a few seconds in a defined area of 2 cm diameter to fully exfoliate the spalled Si film from the c-Si donor substrate. However, the spontaneous spalling process at high annealing temperature occurs with high crack propagation velocity, and usually within a few microseconds (Bellanger et al., 2016; Coll et al., 2018). Arakawa et al. experimentally showed that fractured surface roughness in a brittle material is proportional to crack propagation velocity (Arakawa and Takahashi, 1991). Fineberg et al. showed that low crack propagation velocity results in a smoother fractured surface. In addition, micro-branching could also occur at the fracture surface with high crack propagation velocities (Fineberg et al., 1991; Fineberg and Marder, 1999). The micro-branches at the fracture surface exist as surface defects such as nano/micro-cracks which can significantly reduce the performance of Si photoelectrodes (Dhimish and Holmes, 2019). Therefore, the controlled spalling process conducted at room temperature, which allows controllability over crack propagation velocity through an applied external force, is a better approach to exfoliate thin Si with appropriate material property.

In this spalling process, Ti and Ni were used as adhesion and stressor layers, respectively. The metal elements can diffuse into the spalled Si, and could result in performance degradation (Sakiotis, 1982). Secondary ion mass spectrometry (SIMS) was used to investigate the diffused metal impurity of free-standing spalled Si after the removal of Ti/Ni layers. Figure 4E shows the SIMS depth profile of Ti impurity (see Figure S4 for Ni metal impurity), confirming Ti diffusion of ∼30 nm in depth from the surface when the annealing temperature is lower than 300°C. However, the Ti concentration dramatically increased at an annealing temperature of 400°C and the diffusion depth dramatically increased to ∼500 nm. This region may need to be removed from the spalled Si before fabricating water-splitting photoelectrodes.

Figure 4F shows the processing yield of free-standing spalled Si after removal of the Ni stressor layer. Fail process occurs due to the formation of horizontal cracks in the spalled Si, which subsequently result in the disintegration of the films into multiple pieces after removal of the Ni stressor layer. The processing yields are 67, 25, and 0% for the 25°C, 300°C, and 400°C samples, respectively. We attribute the reduced processing yield at higher annealing temperature to high surface roughness and residual stress on the fracture surface, which result in undesirable cracking states (Echizenya et al., 2011; Yang et al., 2013).

### Application of thin free-standing Si as photoanodes

**Figure 5:**
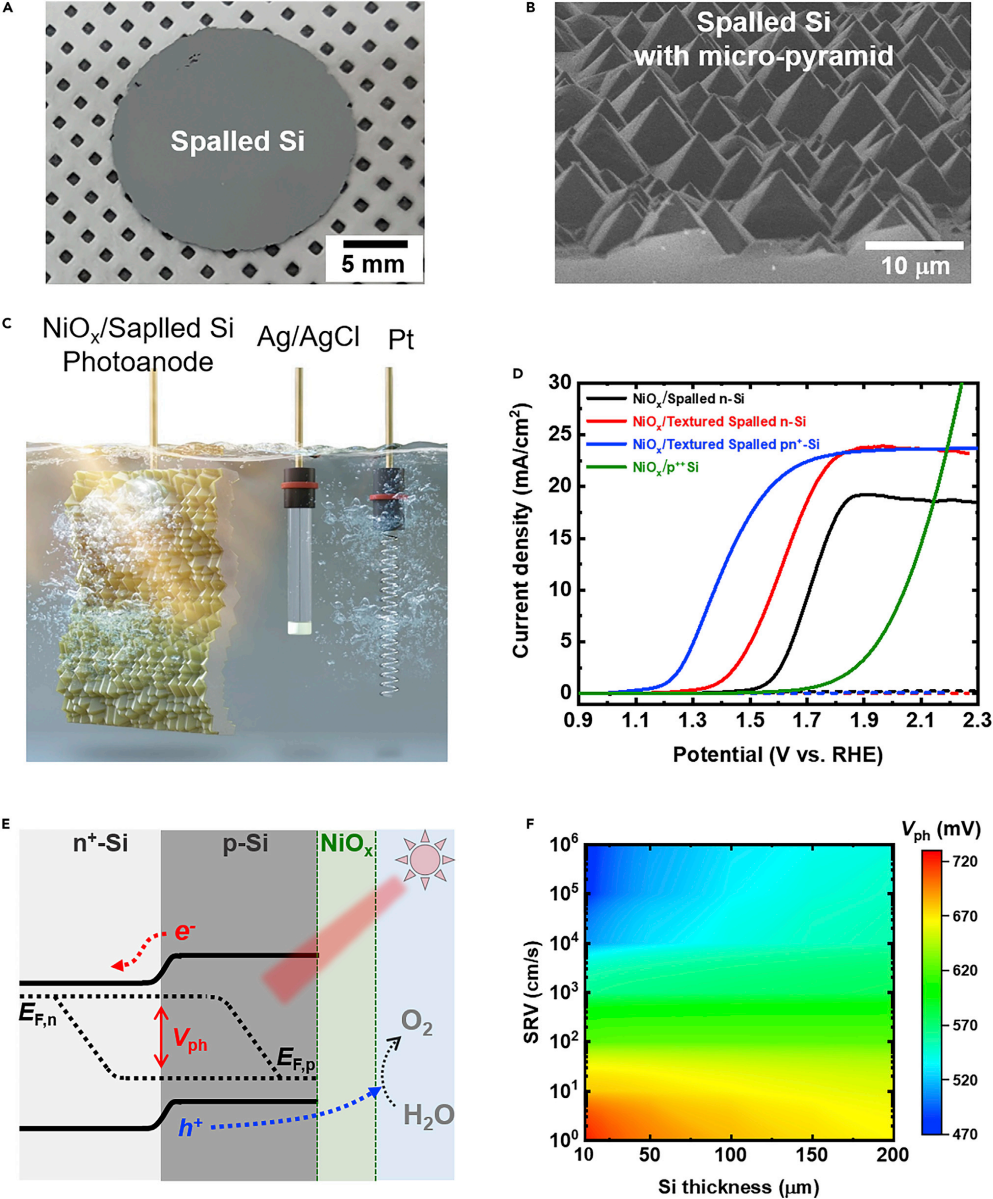
Photoelectrochemical study of ultrathin NiO_x_/Si photoanodes (A) Optical image of a 50 μm-thick spalled Si with a diameter of 2 cm after Ni stressor layer removal. The spalled Si was fabricated by the controlled spalling process using 30 μm-thick Ni based stressor layer at room temperature. (B) Tilted-view SEM image of the spalled Si after texturing with the KOH wet etching process. (C) Artist impression of the PEC cell consisting of ultrathin NiO_x_ -coated spalled Si photoanode in a conventional three electrode configuration. (D) Current density – potential curves of different spalled Si photoanodes. (E) Energy bandgap diagram of NiO_x_-coated spalled Si photoanode with a pn^+^ junction. (F) Calculated photovoltage (*V*_ph_) of spalled Si photoanode with a pn^+^ junction as a function of Si thickness and surface recombination velocity (SRV).

Figure 5A shows free-standing ∼50 μm-thick spalled Si with a diameter of 2 cm produced by the controlled spalling process at room temperature, which presents lowest surface defects after removal of the Ni stressor layer. In addition, the micro-scale pyramid surface structure was obtained using wet-etching process to enhance the photo-generated current density via light scattering effect (see Figure 5B).

Finally, we fabricated and characterized 20 nm-thick NiO_x_-coated spalled Si photoanodes with and without surface texture, labeled as NiO_x_/Spalled n-Si and NiO_x_/Textured Spalled n-Si, in 1M KOH solution using a typical three-electrode configuration under simulated AM1.5G illumination as illustrated in Figure 5C. The NiO_x_ film on Si serves as an efficient OER catalyst, provides surface protection, and could also function as an anti-reflection layer (Sun et al., 2012, 2015). As shown by the linear sweep voltammetry (LSV) measurements in Figure 5D, the planar photoanode shows an onset potential *E*_onset_ of 1.54 V (vs. RHE). It is noted that the *E*_onset_ is defined as the potential required to drive a photo-generated current density of 1 mA/cm^2^. The planar and textured samples show saturation current densities *J*_sat_ of 19.2 and 23.8 mA/cm^2^, respectively. Negligible dark current densities are observed (dotted line in Figure 5D), confirming no undesirable side reactions. Texturing leads to an improvement in current density by 4.6 mA/cm^2^ due to enhanced light trapping compared to the planar photoanode even ∼20 μm of material is removed during the texturing process (see Figure S5). In addition, *E*_onset_ decreases from 1.56 to 1.39 V, showing enhanced photovoltage (*V*_ph_) from the removal of defects on the fracture surface during the surface texturing process (Lee et al., 2018b). This confirms that the surface texturing is crucial to achieve high performance in Si film photoanodes. We also introduced a pn^+^ junction into the spalled Si to increase *V*_ph_. Figure S6 shows the fabrication procedure for the NiO_x_-coated spalled Si photoanode incorporating a rear pn^+^ junction and surface texturing process. As clearly seen in Figure 5D (blue solid line), the sample shows a similar photo-current density but reduced *E*_onset_ due to enhanced *V*_ph_ (0.65 V) from the rear pn^+^ junction compared to the textured sample but without a pn^+^ junction. Here, the *V*_ph_ is approximated as the potential difference between NiO_x_/textured spalled pn^+^-Si and NiO_x_/p^++^-Si at 10 mA/cm^2^ in 1M KOH.

The relatively lower *J*_sat_ value for all the photoelectrodes compared to the theoretically predicted values as presented in Figure 2E is due to undesired light transmission losses in the quartz cell and the electrolyte solution and a lack of surface passivation resulting in charge recombination (see Figure S7) (Lee et al., 2018; Malitson, 1965; Irvine and Pollack, 1968). *J*_sat_ can be further enhanced by including an effective passivation layer and introducing rear surface texturing for light trapping enhancement at long wavelength (Sun et al., 2015; Tamang et al., 2016; Zhang et al., 2014).

Further, we theoretically investigated the effect of passivation on the OER performance. Figure 5E shows the energy band diagram of the NiO_x_-coated spalled photoanode with a rear pn^+^ junction. The splitting of the electron and hole quasi-Fermi level occurs under light illumination, and the difference between the electron and hole quasi-Fermi levels represents *V*_ph_. Figure 5F shows the theoretically calculated *V*_ph_ of spalled Si photoanodes as a function of thickness and surface recombination velocity (SRV). It can be clearly seen that *V*_ph_ reduces dramatically when the thickness of Si is decreased under high SRV (*e*.*g*., >10^3^ cm/s). On the contrary, at low SRV (*e*.*g*., <10 cm/s), *V*_ph_ increases significantly with reducing Si thickness due to increased minority carrier density in the limited light-absorber thickness (Brendel and Queisser, 1993; Zhang et al., 2014). For instance, the calculated *V*_ph_ values at an SRV of 1 cm/s are 665.9., 681.9, 696.8, and 706.5 mV with Si thicknesses of 200, 100, 50, and 30 μm, respectively. As shown above, *V*_ph_ of our NiO_x_/textured spalled pn^+^-Si is 0.65 V and this value is comparable to the theoretically calculated values with Si thickness of 30 μm (0.5–0.7 V). This can be further improved above 0.7 V by incorporating effective surface passivation on thin Si photoanodes leading to superior *V*_ph_ generation compared to conventional wafer-based (∼500 μm) Si photoanodes.

## Discussion

In conclusion, free-standing c-Si films with controlled thickness were fabricated using the spalling process. We systematically investigated the thermal annealing effect on the spalling process and the material properties of the spalled Si. The spalled Si fabricated by the controlled spalling process at room temperature shows relatively low surface roughness, low defect density, and high processing yield compared to that fabricated by the spontaneous spalling process at high annealing temperatures. Finally, we demonstrated NiO_x_-coated spalled Si photoanodes with micro-pyramid texture and a rear pn^+^ junction, which exhibit an *E*_onset_ of 1.2 V and a *J*_sat_ of 23.4 mA/cm^2^. Our work shows that thin free-standing Si photoelectrodes fabricated using crack-assisted exfoliation hold a great potential to achieve cost-effectiveness, enhanced PEC performance, and importantly allow direct integration on curved surfaces.

## Limitations of the study

For high performance Si-based photoanodes, it is crucial to apply efficient co-catalysts with high activity for the OER. In this study involving a proof-of-concept demonstration, we applied transparent NiO_x_ as oxygen evolution catalyst for the evaluation of the PEC water splitting performance on thin Si-based photoelectrode. The performance of our thin Si-based photoelectrodes can be further significantly improved by incorporating multimetallic/multi-component co-catalysts and adopting a decoupled photoelectrode design which separates the light harvesting from the catalytic interface.

## STAR★Methods

### Key resources table

**Table:** 

REAGENT or RESOURCE	SOURCE	IDENTIFIER
**Chemicals**		

Nickelchloride hexahydrate	Sigma-Aldrich	CAS: 7791-20-0
Boric acide	Sigma-Aldrich	CAS: 10043-35-3

**Software and algorithms**		

COMSOL Multiphysics	COMSOL, Inc.	Version 5.4

**Other**		

Potentiostat	CH Instruments, Inc.	CHI660E
Illuminaiton source	ABET Technology	model 11002-2

### Resource availability

#### Lead contact

Future information and requests for resources should be directed to and will be fulfilled by the lead contact, Yonghwan Lee (yhlee@geri.re.kr)

#### Materials availability

New unique reagents were not generated in this study.

#### Data and code availability

Data and code would be made available upon request.

### Method details

#### Spalling process for Si film exfoliation

Mirror-polished 500 μm-thick n-type Czockraski (CZ) c-Si (100) wafers (phosphorus doped, *ρ* = 1-10 Ω cm) were used as c-Si donor substrates. First, the c-Si donor substrates were cleaned by RCA 1 process, followed by dipping in a diluted HF (5 wt%) solution to remove the native oxide. Subsequently, a bilayer consisting 50 nm Ti (adhesion layer) and 50 nm Ni (seed layer) was deposited on the Si substrates using e-beam evaporator prior to Ni stressor layer deposition using electrodeposition process. A mask with a circular pattern of 2 cm diameter was attached onto the Ni/Ti coated Si substrates to define the electrodeposition area. The substrates were dipped in a diluted HCl (10 wt%) solution for 5 min, followed by rinsing in deionized water. The Ni electrodeposition was conducted in a chlorine-based Ni solution. After the Ni electroplating process, the spalling process was conducted via two different approaches, namely, spontaneous spalling and controlled spalling. For the former method assisted by a thermal annealing process, the Ni/Ti/Si substrates were annealed using a rapid thermal annealing (RTA) system for 5 min under varying annealing temperatures (300–400°C) in Ar atmosphere. After cooling-down in the RTA system, Si layers were spontaneously exfoliated due to the propagation of a sub-surface crack. For the controlled spalling process, an initial sub-surface crack was formed by mechanical wedge at/near the edge of Ni stressor layer and the locally separated Si was grasped by a tweezer and pulled in a vertical direction. Subsequently, the spalled Si was completely peeled away from the c-Si donor substrates. After the spalling process, the Ni stressor layer attached to the spalled Si was removed by wet etching in HCl/H_2_O_2_/H_2_O mixed solution (3:3:10, v/v/v) to produce free-standing Si.

#### Numerical optical simulations of Si film photoanodes

Light absorption and photocurrent density of c-Si substrates were simulated using Wafer Ray Tracer (WRT) simulation software (Version 1.6.7, PV Lighthouse Pty. Ltd., Australia) at 20 nm intervals and 5000 incident rays with different Si thickness (PV Lighthouse: Wafer Ray Tracer, 2016). The surrounding material was set to H_2_O. The incident sunlight was chosen to be AM1.5G and the calculations were performed in the wavelength range of 300–1200 nm. The refractive indices of the materials used in the simulation were taken from the literature (Green, 2008). The two-dimensional electrical field distribution and absorption profiles were analyzed using the wave optics module of COMSOL Multiphysics 5.4 software package. The detailed simulation structures are described in Figure S1. The absorption profile was calculated with electric field *E*(λ) at specific wavelengths (*λ)* using the following equation:

(Equation 2) $$P_{abs}\left(\lambda \right)=\;\frac{1}{2}\omega \varepsilon^{"}{\left|E\left(\lambda \right)\right|}^{2}$$

where *ω* is angular frequency which is equal to 2π/λ and $\varepsilon^{"}$ is the imaginary component of complex permittivity of the material (Bednar et al., 2015).

#### Materials characterization

The thicknesss of spalled Si layers were measured using field-emission scanning electron microscopy (FE-SEM, Nova230, FEI Co., USA). The surface profile and roughness were characterized by surface profilometry (Dektak-8, VEECO, USA). The residual stress at the fracture surface of spalled Si was measured by micro-Raman spectrometer (DXR, Thermo Fisher Scientific, USA) with a laser wavelength of 514 nm. The stress *σ*_Si_ at the fracture surface in free-standing spalled Si was calculated using the following equation:

(Equation 3) $$\sigma_{Si,xx}=\sigma_{Si,yy}\left(\text{MPa}\right)=\;-250\times \Delta \omega \;\left(cm^{-1}\right).$$

where *Δω* is the Raman peak position shift between unprocessed c-Si donor substrate and free-standing spalled Si (Farrukh, 2012). Secondary ion mass spectrometry (SIMS, IMS 7f, CAMECA, France) was used to investigate the diffused metal impurities at the surface of free-standing spalled Si after the removal of the Ni stressor layer.

#### Fabrication of *NiO*_x_-coated spalled Si photoanodes

To prepare pyramidal structures on the spalled Si, the 50 μm-thick free-standing Si obtained via the controlled spalling at room temperature was immersed into a mixed solution of KOH, isopropyl alcohol, and H_2_O at 70–80°C for 40 min, followed by a cleaning step in HCl/H_2_O_2_/H_2_O mixed solution (1:1:5, v/v/v)) at ∼80°C for 10 min. The cleaned spalled Si was immersed in a diluted HF (10 wt%) solution for 1 min to remove the surface native oxide. A 20 nm thick NiO_x_ cocatalyst film was deposited on the surface of the spalled Si using DC sputtering technique (NiO sputtering target, Ar atmosphere). An ohmic rear contact was formed using an In-Ga eutectic alloy and a copper wire was attached to this contact. In order to isolate and protect the rear contact of the spalled Si photoanodes in electrolyte solutions, the samples were sealed off using an epoxy (Loctite 9460), leaving the front surface (with the NiO_x_ cocatalyst film) for direct contact with the solution to participate in OER under illumination.

#### PEC measurement of spalled Si photoanodes

The PEC properties of the spalled Si were characterized in a typical three-electrode measurement configuration using a potentiostat. A Ag/AgCl (3M NaCl) and a Pt coil were used as the reference and counter electrodes, respectively. The illumination source was a 300 W Xe lamp (ABET Technology, model 11,002-2) equipped with AM1.5G filters. Light intensity was set to 1 sun using a reference solar cell. All the PEC polarization curves (*J*-*E*) were recorded in the anodic direction at a scan rate of 20 mV/s in 1 M KOH solution. All potentials were expressed as reversible hydrogen electrode (RHE) by *E*_RHE_ = *E*_WE_ + (0.209 + 0.059×pH) V.

#### Fabrication of NiO_x_/Textured spalled pn^+^-Si photoanodes

A lightly doped 500 μm-thick p-type Czockraski Si (boron-doped, 1-10 Ω cm) wafers (100) were used. An n^+^ region was formed by phosphorus implantation with a dose of 1.2×10^15^ cm^−2^ on top of a p-type Si wafer at an acceleration voltage of 80 keV. Afterward, an annealing process at 1000°C for 30 s in nitrogen (N_2_) atmosphere was conducted to cure the damages induced by the ion implantation process. Subsequently, a Ti/Ni (50/50 nm) layer was deposited over the n^+^ region of Si substrate using e-beam evaporator followed by Ni electrodeposition process (∼30 μm thickness). Controlled spalling process was conducted at room temperature to exfoliate a Si film with the rear pn^+^ junction and immersed into the KOH/IPA/H_2_O mixed solution at 70–80°C for 40 min for surface texturing. The Ni stressor layer and e-beam evaporated Ti/Ni layer was removed by a HCl/H_2_O_2_/H_2_O mixed solution followed by diluted HF treatment. The 20 nm-thick NiO_x_ film was deposited on the surface of the spalled Si using DC sputter. Ohmic contact was formed at the n^+^ region using In-Ga eutectic alloy which was further connected to a copper wire. Finally, the sample was sealed with epoxy, leaving only the NiO_x_ surface in contact with the electrolyte.

#### Photovoltage calculation of spalled Si with pn^+^ junction

The photovoltage *V*_ph_ of the spalled Si with a rear pn^+^ junction was calculated as a function of SRV and Si thickness using the following equations:

(Equation 4) $$V_{ph}=\frac{kT}{q}\;\ln\left(\;\frac{J_{L}}{J_{0}}+1\right)$$

where *k* is the Boltzmann constant, *T* is absolute temperature, *q* is the electron charge, *J*_L_ is the photo-current density, and *J*_0_ is the diode saturation current density (Green, 1982). *J*_0_ was calculated as:

(Equation 5) $$J_{0}=\left(\frac{qD_{e}n_{i}^{2}}{L_{e}N_{A}}F_{P}+\;\frac{qD_{h}n_{i}^{2}}{L_{h}N_{D}}F_{N}\right)$$

where *D*_e_ is electron diffusivity in p-type region, *D*_h_ is hole diffusivity in n-type region, *n*_i_ is the intrinsic carrier concentration, *L*_e_ is electron diffusion length in p-type region, *L*_h_ hole diffusion length in n-type region, *N*_A_ is acceptor doping concentration in p-type region, and *N*_D_ is donor doping concentration in n-type region. It is noted that the substrate boron doping concentration was calculated using the resistivity calculator by setting the resistivity to 5 Ω·cm (PV Lighthouse: Resistivity Calculator, 2020). *L*_e_, *L*_h_, *F*_N_ and *F*_P_ were calculated using the following equations:

(Equation 6) $$L_{e}=\;\sqrt{D_{e}\tau_{e}\;}$$

(Equation 7) $$L_{h}=\;\sqrt{D_{h}\tau_{h}\;}$$

(Equation 8) $$F_{N}=\frac{S_{h}\;\cosh\left(\frac{W_{N}}{L_{h}}\right)+\;\frac{D_{h}}{L_{h}}sinh\left(\frac{W_{N}}{L_{h}}\right)}{\frac{D_{h}}{L_{h}}cosh\left(\frac{W_{N}}{L_{h}}\right)+\;S_{h}\;\sinh\left(\frac{W_{N}}{L_{h}}\right)}$$

(Equation 9) $$F_{P}=\frac{S_{e}\;\cosh\left(\frac{W_{P}}{L_{e}}\right)+\;\frac{D_{e}}{L_{e}}sinh\left(\frac{W_{P}}{L_{e}}\right)}{\frac{D_{e}}{L_{e}}cosh\left(\frac{W_{P}}{L_{e}}\right)+\;S_{e}\;\sinh\left(\frac{W_{P}}{L_{e}}\right)}$$

where *τ*_e_ is the electron carrier lifetime time in p-type region, *τ*_h_ is the hole carrier lifetime in n-type region, *S*_e_ is the SRV in p-type region, and *S*_h_ is SRV in n-type region, *W*_p_ is the p-type region width, and *W*_n_ is the n-type region width. It is noted that the n- and p-type region width (junction depth) were calculated using a ion implantation calculator (Diffused Ion Implantation Profile Calculator and Graph, 2020). The respective values of the parameters used in the calculations are summarized in Table S1 in the supplemental information.

## Footnotes

Supplemental information can be found online at https://doi.org/10.1016/j.isci.2021.102921.
