# Supplementary material for: Thin silicon via crack-assisted layer exfoliation for photoelectrochemical water splitting
Source: iScience. 2021 Jul 30;24(8):102921. doi: 10.1016/j.isci.2021.102921 (PMC8367840; doi:10.1016/j.isci.2021.102921)
Supplement: Document S1. Figures S1–S8 and Table S1 [file mmc1.pdf]

iScience, Volume *24*

Supplemental information

Thin silicon via crack-assisted layer exfoliation for photoelectrochemical water splitting

Yonghwan Lee, Bikesh Gupta, Hark Hoe Tan, Chennupati Jagadish, Jihun Oh, and Siva Karuturi

Supplemental Information

Thin Silicon via Crack-Assisted Layer Exfoliation for Photoelectrochemical Water Splitting

Yonghwan Lee, [1,2,3,*] *Bikesh Gupta,* [1] *Hark Hoe Tan,* [1,4] *Chennupati Jagadish,*[1,4] *Jihun Oh,*[5] *and Siva Karuturi*[1, 6,*]

[1]Department of Electronic Materials Engineering, Research School of Physics, The Australian National University, Canberra, ACT 2601, Australia

[2]Convergence Materials Research Center, Gumi Electronics and Information Technology Research Institute (GERI), Gumi 39171, Republic of Korea

[3]Lead Contact

[4]Australian Research Council Center of Excellence for Transformative Meta-Optical Systems, Research School of Physics, The Australian National University, Canberra, ACT 2601, Australia

[5]Department of Materials Science and Engineering, Korea Advanced Institute of Science and Technology (KAIST), Daejeon 34141, Republic of Korea

[6]Research School of Engineering, The Australian National University, Canberra, ACT 2601, Australia

Correspondence: yhlee@geri.re.kr; siva.karuturi@anu.edu.au

Keywords: crystalline silicon, thin semiconductor, controlled-cracking, photoanode, water splitting

Table S1. Parameters used for photovoltage calculation of spalled Si with a rear pn^+ junction, related to STAR Methods.

**Symbol**	**Description**	**Value**
n_i	Intrinsic carrier concentration	1.5×10^{10} cm^{-3}
q	Electronic charge	1.6×10^{-19} C
k	Boltzmann constant	8.62×10^{-5} eV/K
T	Absolute temperature	300 K
τ_e	Electron bulk lifetime in p-type region	1 ms
τ_h	Hole bulk lifetime in n-type region	1 ms
N_A [1)]	Doping concentration in p-type region	2.76×10^{15} cm^{-3}
N_D [2)]	Doping concentration in n-type region	1×10^{18} cm^{-3}
D_e	Electron diffusivity in p-type region	33.74 cm^2/s
D_h	Hole diffusivity in n-type region	11.98 cm^2/s
W_N [2)]	N-type region width	0.3 μm
$\mathrm{W_P}$	P-type regions width	Variable
S_e	Surface recombination velocity in p-type region	Variable
S_h	Surface recombination velocity in n-type region	Variable (Same with the S_e)

[1)]Value extracted from the resistivity calculator by setting the resistivity of c-Si substrate to 5 Ω·cm.

[2)]Value extract from the ion implantation calculator with the condition of the ion-implantation process (see Figure S8)

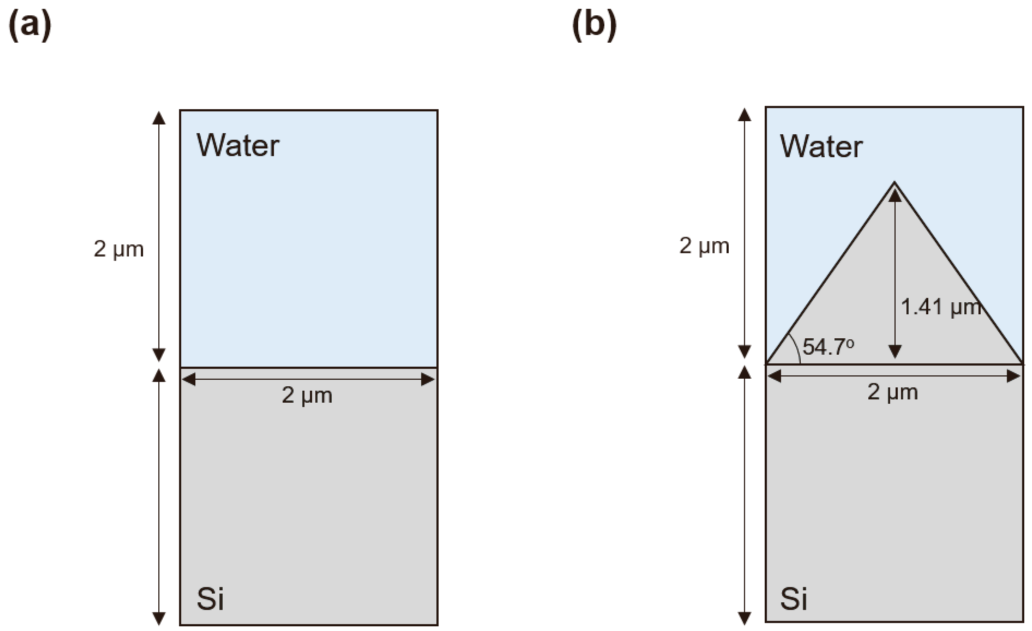

Figure S1. Schematic illustration of the geometry used for finite-element method (FEM) optical simulation, related to Figure 2. (a) Planar Si and (b) textured Si.

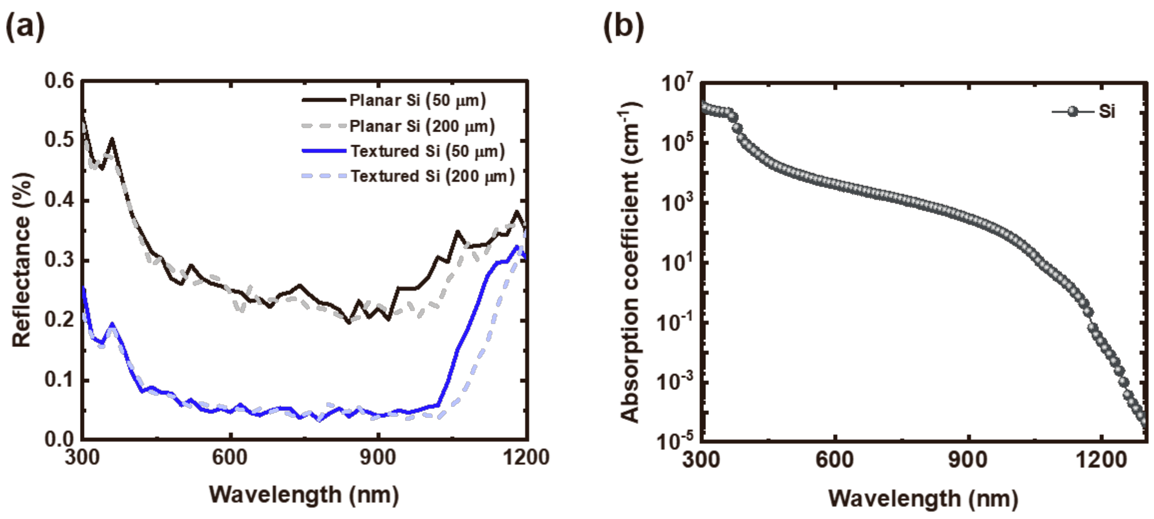

Figure S2. Simulated reflectance and absorption coefficient on Si, related to Figure 2. (a) Simulated reflectance on the spalled Si with (blue plots) and without texture (black plots) for two different thicknesses using Wafer Ray Tracer software ((PV Lighthouse: Wafer Ray Tracer, 2016). (b) Absorption coefficient α in c-Si as a function of wavelength λ. α was calculated with the equation: $\alpha = 4\pi k/\lambda$, where k is the extinction coefficient. The k value is used from reference. (Green, 2008)

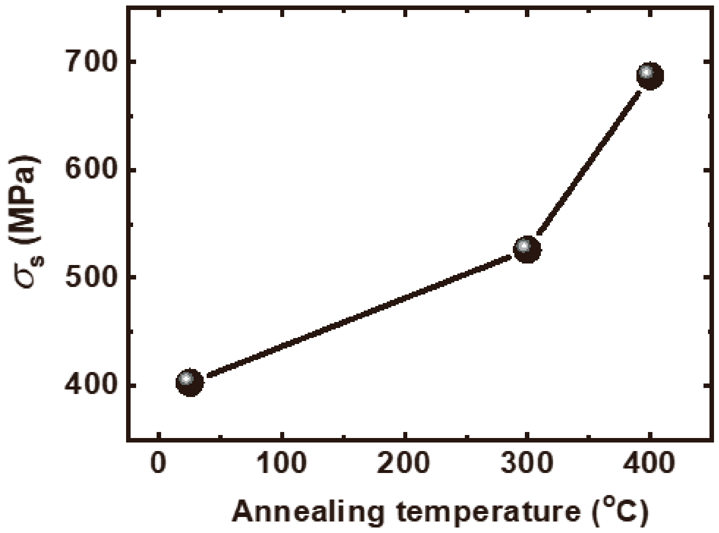

Figure S3. Residual stress of Ni stressor layer on c-Si substrate as a function of annealing temperature measured by X-ray diffraction $\sin^2(\Psi)$ technique, related to Figure 3. (Noyan and Cohen, 2013) For the measurements, 10 μm-thick Ni stressor layer on the c-Si substrate was used.

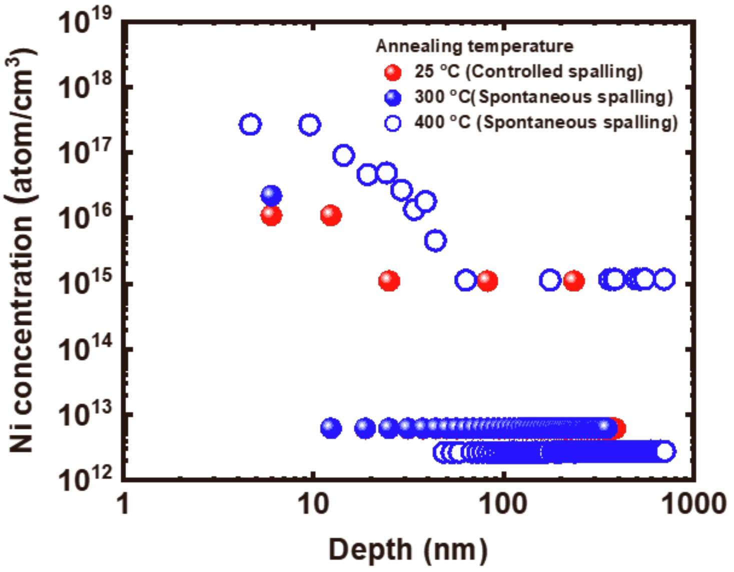

Figure S4. Secondary ion mass spectrometry (SIMS) profile of Ni impurity in the spalled Si for different annealing temperatures, related to Figure 4. The measurements were conducted after removal of the Ni stressor layer from the spalled Si.

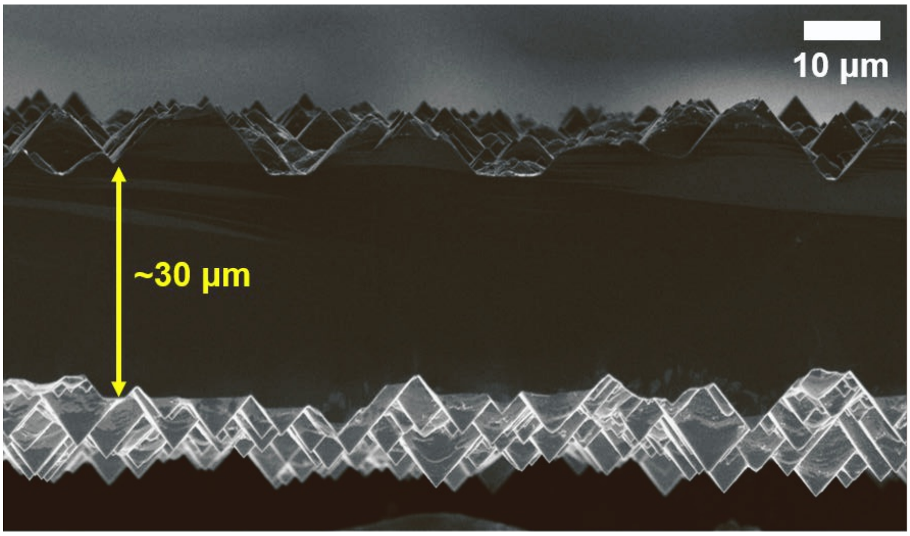

Figure S5. Cross-sectional scanning electron micrograph of the spalled Si after wet etching process for surface texturing, related to Figure 5. The wet etching process was conducted in mixed solution of KOH, isopropyl alcohol, and H_2O at 70 – 80 ℃ for 40 min

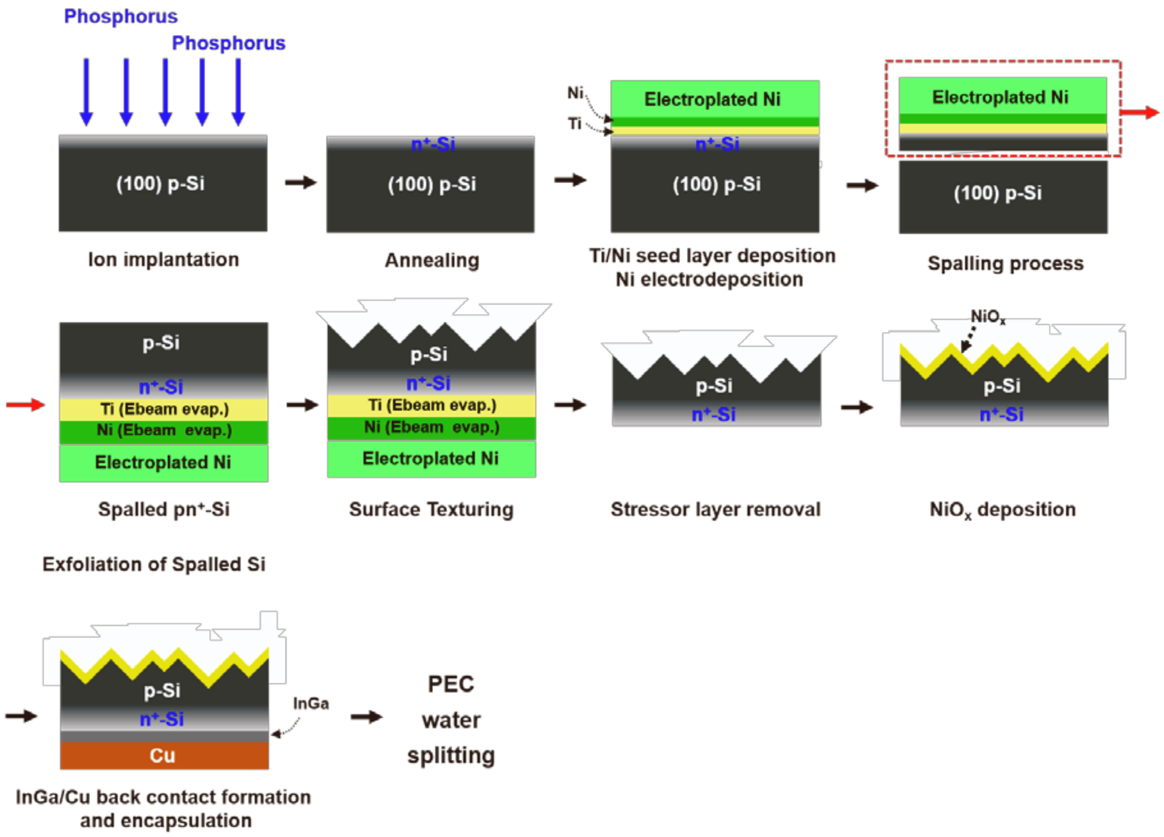

Figure S6. Schematic illustration of the fabrication process for the NiO_x/Textured Spalled pn^+-Si photoanodes for PEC water splitting, related to Figure 5. A lightly doped 500 μm-thick p-type Czockraski Si (boron-doped, 1-10 Ω cm) wafers (100) were used. An n^+ region was formed by phosphorus implantation with a dose of 1.2×10^{15} cm^{-2} on top of a p-type Si wafer at an acceleration voltage of 80 keV. Afterward, an annealing process at 1000 ℃ for 30 sec in nitrogen (N_2) atmosphere was conducted to cure the damages induced by the ion implantation process. Subsequently, a Ti/Ni (50/50 nm) layer was deposited over the n^+ region of Si substrate using e-beam evaporator followed by Ni electrodeposition process (~30 μm thickness). Controlled spalling process was conducted at room temperature to exfoliate a Si film with the rear pn^+ junction and immersed into the KOH/IPA/H_2O mixed solution at 70 – 80 ℃ for 40 min for surface texturing. The Ni stressor layer and e-beam evaporated Ti/Ni layer was removed by a $HCl/H_2O_2/H_2O$ mixed solution followed by diluted HF treatment. The 20 nm-thick NiO_x film was deposited on the surface of spalled Si using DC sputter. Ohmic contact was formed at the n^+ region using In-Ga eutectic alloy which was further connected to a copper wire. Finally, the sample was sealed with epoxy, leaving only the NiO_x surface in contact with the electrolyte.

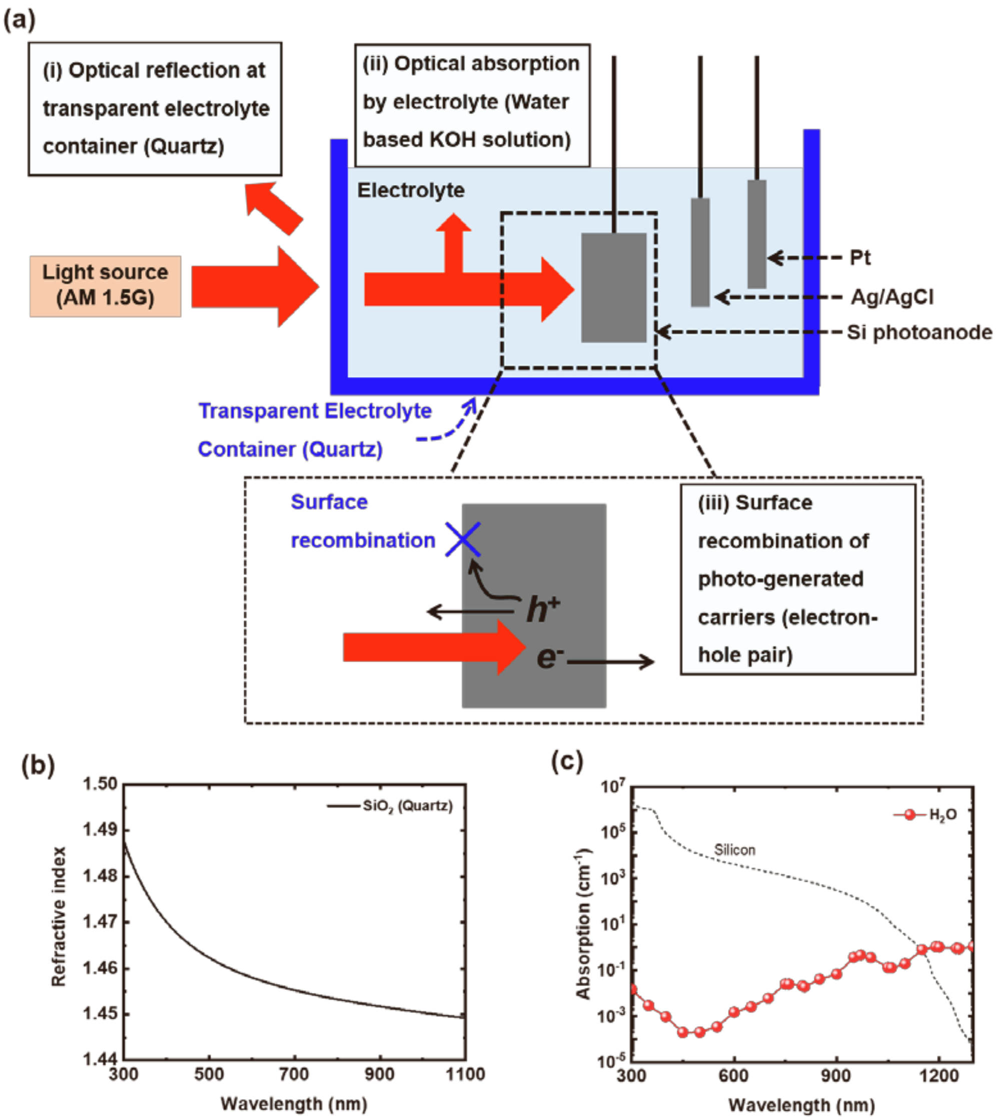

Figure S7. The mian causes for the reduced photo-generated current density values of spalled Si based photoanodes compared to the theoretically calculated values, related to Figure 5. (a) Schematic illustration of the main causes for the reduced photo-generated current density values of spalled Si based photoanodes compared to the theoretically calculated values in Fig. 2e. (b) Refractive index of quartz as a function of wavelength.(Malitson, 1965) The optical reflection of incident solar radiation occurs due to a difference in refractive indices of air and quartz. (c) Absorption coefficient of water molecules (H_2O). (Irvine and Pollack, 1968) The dotted line is the absorption coefficient of Si. The electrolyte consists of aqueous solution which absorbs the incident solar radiation especially at long wavelength.

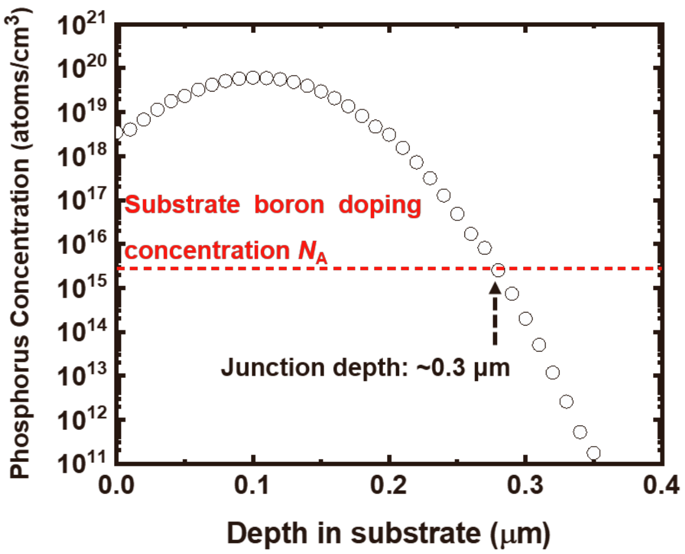

Figure S8. Calculated phosphorus doping concentration and junction depth by ion-implantation process using the ion implantation calculator, related to STAR Methods (Diffused Ion Implantation Profile Calculator and Graph, 2020). In the calculation, ion implantation process conditions mentioned in Figure S6 were used. The substrate boron doping concentration was calculated using the resistivity calculator by setting the resistivity to 5 Ω·cm (PV Lighthouse: Resistivity Calculator, 2020).
